# Development of nuclear microsatellite loci for *Pinus albicaulis* Engelm. (Pinaceae), a conifer of conservation concern

**DOI:** 10.1371/journal.pone.0205423

**Published:** 2018-10-18

**Authors:** Marian V. Lea, John Syring, Tara Jennings, Richard Cronn, Leo P. Bruederle, Jennifer Ramp Neale, Diana F. Tomback

**Affiliations:** 1 Department of Integrative Biology, University of Colorado Denver, Denver, Colorado, United States of America; 2 Department of Biology, Linfield College, McMinnville, Oregon, United States of America; 3 Pacific Northwest Research Station, United States Department of Agriculture, Forest Service, Corvallis, Oregon, United States of America; 4 Denver Botanic Gardens, Denver, Colorado, United States of America; Technical University in Zvolen, SLOVAKIA

## Abstract

*Pinus albicaulis* (whitebark pine) is a widely-distributed but rapidly declining high elevation western North American tree and a candidate for listing under the U.S. Endangered Species Act. Our objectives were to develop reliable nuclear microsatellite markers that can be used to assess within-population genetic diversity as well as seed and pollen migration dynamics, and to validate markers using two geographically proximal *P*. *albicaulis* populations. We identified 1,667 microsatellite-containing sequences from shotgun DNA libraries of *P*. *albicaulis*. Primer pairs were designed for 308 unique microsatellite-containing loci, and these were evaluated for PCR amplification success and segregation in a panel of diploid needle tissue. DNA was extracted with an SDS protocol, and primers were screened through gel electrophoresis. Microsatellites were genotyped through fluorescent primer fragment analysis. Ten novel and 13 transferred loci were found to be reproducible in analyses based on 20 foliage samples from each of two locations: Henderson Mountain, Custer Gallatin National Forest, Montana, and Mt. Washburn, Yellowstone National Park, Wyoming (USA). Transferred loci had higher numbers of alleles and expected heterozygosities than novel loci, but also revealed evidence for a higher frequency of null alleles. Eight of the 13 transferred loci deviated significantly from Hardy-Weinberg Equilibrium, and showed large positive F_IS_ values that were likely inflated by null alleles. Mantel’s tests of transferred and novel markers showed no correlation between genetic and geographic distances within or among the two sampled populations. AMOVA suggests that 91% of genetic variability occurs within populations and 9% between the two populations. Studies assessing genetic diversity using these microsatellite loci can help guide future management and restoration activities for *P*. *albicaulis*.

## Introduction

North American forest trees have been exposed to a number of unprecedented health challenges that are likely to be exacerbated by warming temperatures [1]. These challenges include outbreaks of native insects and especially bark beetles [2], mortality of older age classes from drought [3], and the accidental introduction and spread of destructive exotic pests and pathogens (e.g., [4]). In fire-prone communities in the western United States, intervals between fires are projected to decline over the next century, as climate warms [5, 6], and this may lead to metapopulation extirpation and the extinction of some woody plant species [7, 8]. As populations regenerate following disturbance, the recovery of community structure and genetic diversity is extremely important [9, 10].

*Pinus albicaulis* Engelm. (Pinaceae, whitebark pine) is a widely distributed western North American tree species of subalpine and treeline elevations ranging from about 37° to 55° N latitude. It functions as an ecological keystone and foundation species in these high elevation communities [11, 12]. The species, however, has experienced range-wide mortality due to insect outbreaks, pathogens, and climate change; consequently, it is a candidate for listing under the U.S. Endangered Species Act [13]. *Pinus albicaulis* is a slow-growing species that does not produce large cone crops until at least 60 years of age [14]. It tolerates cold, dry, and windy environments, forming self-replacing stands on harsh sites. On productive sites, *P*. *albicaulis* is an early seral species in successional communities, which depend on disturbance, primarily fire, for renewal (e.g., [15]). It relies on the Clark’s nutcracker (*Nucifraga columbiana* Wilson) for seed dispersal, which influences its ecology and population biology [16–19]. *Pinus albicaulis* regenerates rapidly from seed caching in burned terrain by nutcrackers following fires, with new seedlings establishing within a few years [20–22]. At treeline elevations on arid sites, it is often dominant and a tree island initiator, facilitating infilling and upward expansion of the alpine treeline ecotone [23–25]. *Pinus albicaulis* treeline communities redistribute and retain snow, regulating downstream flows in summer. In addition, the large seeds comprise an important wildlife food source for many granivorous birds besides Clark’s nutcrackers, as well as small mammals and bears [26].

*Pinus albicaulis* is a host for mountain pine beetles (*Dendroctonus ponderosae* Hopkins), which have erupted in large-scale outbreaks in the Rocky Mountains and elsewhere in the West over the past 20 years. It is also susceptible to white pine blister rust, which is caused by the introduced pathogen *Cronartium ribicola* J.C. Fisch. and continues to spread geographically [22, 27, 28]. Both tree mortality from mountain pine beetles and mortality and canopy damage from blister rust progressively reduce the number of cone-producing individuals, depleting genetic diversity for future generations [2]. Blister rust also infects and kills young trees, thereby reducing regeneration [29]. In *P*. *albicaulis*, some evidence suggests that heterozygotes are more tolerant of blister rust [30]. However, with declines in cone production and high mortality, heavily impacted *P*. *albicaulis* stands may be experiencing various degrees of inbreeding or pollen limitation. Given decades of decline, *P*. *albicaulis* has become a species of management and conservation concern for federal agencies in the U.S. and Canada [27, 31, 32]. Developing cost-efficient methods to identify the magnitude and apportionment of genetic diversity in *P*. *albicaulis* populations is a high priority, since this can expedite conservation actions. These actions potentially include tracking parentage to search for pest and pathogen resistance, assessment of local genetic diversity to highlight populations in need of additional protection, restoration of post-fire genetic diversity to pre-burn levels, and to inform decisions on seed collections (e.g., [33, 34]).

Nuclear microsatellites (nSSRs) have been a key tool for estimating genetic diversity in species of conservation concern. They have been used to differentiate among populations (pequi, *Caryocar brasiliense* Camb.) and monitor hybridization between rare and widespread taxa (Colorado hookless cactus, *Sclerocactus glaucus* [K. Schumann] L.D. Benson) [35, 36]. They have also been used successfully to describe genetic diversity in threatened conifers [37–41]. Genetic diversity in *P*. *albicaulis* has been previously estimated using genetic markers other than nSSRs, including allozymes [42, 43], organellar simple sequence repeats [34, 44], and single nucleotide polymorphisms (SNPs) [45–47]. However, nSSRs are more accurate and efficient for studies focusing on individual-level differences, including research on sibling reconstruction, linkage disequilibrium, genetic structure, parentage, and individual identification [48]. Thus, nuclear microsatellites should be a useful tool to characterize population genetic diversity and structure in *P*. *albicaulis* and to monitor spatial and temporal changes in genetic composition within stands and across populations. Despite the utility of nSSRs for surveys of genetic diversity in species of conservation concern, nuclear microsatellites have not yet been successfully developed for *P*. *albicaulis*. The species has a large, highly repetitive genome that has confounded efforts to develop single locus polymorphic microsatellites [49]. In addition, microsatellite transfer among pine species has been inconsistent [50].

To facilitate finding useful nSSR markers, many investigators look to near-relatives, such as congeneric taxa including sister species for potential transfer [40, 51–53]. *Pinus albicaulis* is one of 21 pines in section *Quinquefoliae* subsection *Strobus* [54, 55], which in the most recent classification includes the stone pines of former subsection *Cembrae* [56, 57]. Divergence dates for subsection *Strobus* (based on nuclear molecular clock estimates; [58]) are estimated at 11–20 mya. The most recently published phylogenies for *Pinus* subgenus *Strobus* constructed from nuclear, mitochondrial, and chloroplast gene sequences variously resolve *P*. *albicaulis* with species of subgenus *Strobus* native to North America, Asia, and Europe [59].

Here, our first and primary objective was to develop reliable nSSR markers in *P*. *albicaulis* in order to provide an efficient, informative tool to monitor genetic diversity of populations in light of current and future threats. Our secondary objective was to determine the utility of these nSSR markers by comparing genetic diversity in two populations previously studied in the Greater Yellowstone Ecosystem [43]. To maximize the number of nSSRs, we combined screening of previously identified loci from related pine species for potential transferability with surveying *P*. *albicaulis* shotgun nuclear genomic sequences for taxon-specific SSR sequences.

## Materials and methods

To develop novel microsatellite loci for *P*. *albicaulis*, we identified di- and tri-nucleotide repeats using the procedure outlined in Jennings et al. [60], a strategy that was designed to identify very short (< 80bp) paired-end sequence data from early-generation Illumina sequencers. Genomic DNA from diploid needle tissue from a single tree (Custer Gallatin National Forest, Montana; 45.443°N, -110.005°W, 2,470 m a.s.l.) was used to make a standard Illumina library containing internal ‘barcoding’ adapters (barcode 5’-CACT; [61]). This library was pooled with 15 additional barcoded libraries from other conifer species, and five pmol were sequenced on an Illumina Genome Analyzer II using 80 bp paired end reads (University of Oregon Genomics Core Facility, Eugene, OR, USA). Libraries were sorted by barcode using a custom Perl script (https://github.com/listonlab/utility-scripts/blob/master/bcsort_v5.pl). For this strategy, paired microreads are concatenated and separated by 50 N’s (e.g., forward read + 50 N’s + reverse complement reverse read), and identical to nearly-identical sequences (> 95% identity) are filtered to a single representative sequence using cd-hit-454 (Niu et al. 2010). This allowed us to search for dinucleotide (>5 repeats) and trinucleotide (>4 repeats) motifs in the Read 1 or Read 2 sequences using SSR_pipeline [62], and to design flanking amplification primers in the Read 1 and Read 2 sequences using BatchPrimer3 [63] and synthesized at Integrated DNA Technologies (IDT, Coralville, IA, USA). We used default settings for BatchPrimer3, with the exception of the following parameters: product length (min = 100 bp, max = 200 bp), primer length (min = 17 bp, max = 25 bp, optimum = 19 bp), melting temperature (min = 48°C, max = 63°C, optimum = 54°C); number of primers per sequence = 1. The original sequencing reads are deposited at the NCBI Short Read Archive under accession SRR7944190. Primers for 49 nSSR loci were screened for transferability to *P*. *albicaulis*, including 14 loci from *P*. *cembra* L. (Swiss stone pine) [64, 65], three loci from *P*. *parviflora* (Japanese white pine) [66], 13 loci from *P*. *koraiensis* Siebold & Zucc. (Korean pine) [67], and 19 loci from *P*. *strobus* L. (eastern white pine) [68] (S1 Table).

DNA was extracted from silica-dried needles using an SDS-based protocol optimized for *Pinus* species, modified with 1μl β-mercaptoethanol per 404μl of grinding buffer [69]. DNA was eluted in 130μl TE buffer, and concentrations were determined using a NanoDrop 2000 microspectrophotometer (Thermo Scientific, Wilmington, DE, USA). Microsatellite screening used 10μl polymerase chain reaction (PCR) mixtures containing 1X GoTaq G2 Master Mix (Promega, Madison, WI, USA), BSA (1 μM), mixed forward and reverse primers (2.5 μM), and 10ng DNA. Amplifications for novel primers were performed in an Eppendorf vapo.protect Mastercycler proS using the following cycling conditions: denaturation for 5 min. at 95°C, 35 cycles of 45 s each at 95°C, 45 s at 58°C, and 15 s at 72°C, followed by a final annealing cycle of 72°C for 5 min. Transferred primers were amplified using the conditions described in the literature [64–68]. PCR products were visualized on 2% agarose gel using GelRed (Biotium, Fremont, CA, USA) and a low molecular weight DNA ladder (New England BioLabs, Ipswich, MA, USA).

Fluorescent primers were designed with 5’ tags [70]. Microsatellite locus amplifications for fragment analysis were created using the Fluorescent Tag Microsatellite PCR Protocol, with minor modifications. Reactions consisted of 10.5μl mixtures containing 1X GoTaq G2 Colorless Master Mix (Promega, #M7832), BSA (1 μM), non-tagged primer (5 μM), tagged primer (0.5 μM), fluorescent tag (5 μM; VIC, PET, or FAM), and 10ng DNA using the above conditions. PCR products were mixed in multiplexes of three and sent to the DNA Laboratory at Arizona State University for fragment analysis (School of Life Sciences, Tempe, AZ, USA). Fragment analysis outputs were visualized and genotyped using *GeneMarker* V2.6.2 (SoftGenetics).

**Fig 1:**
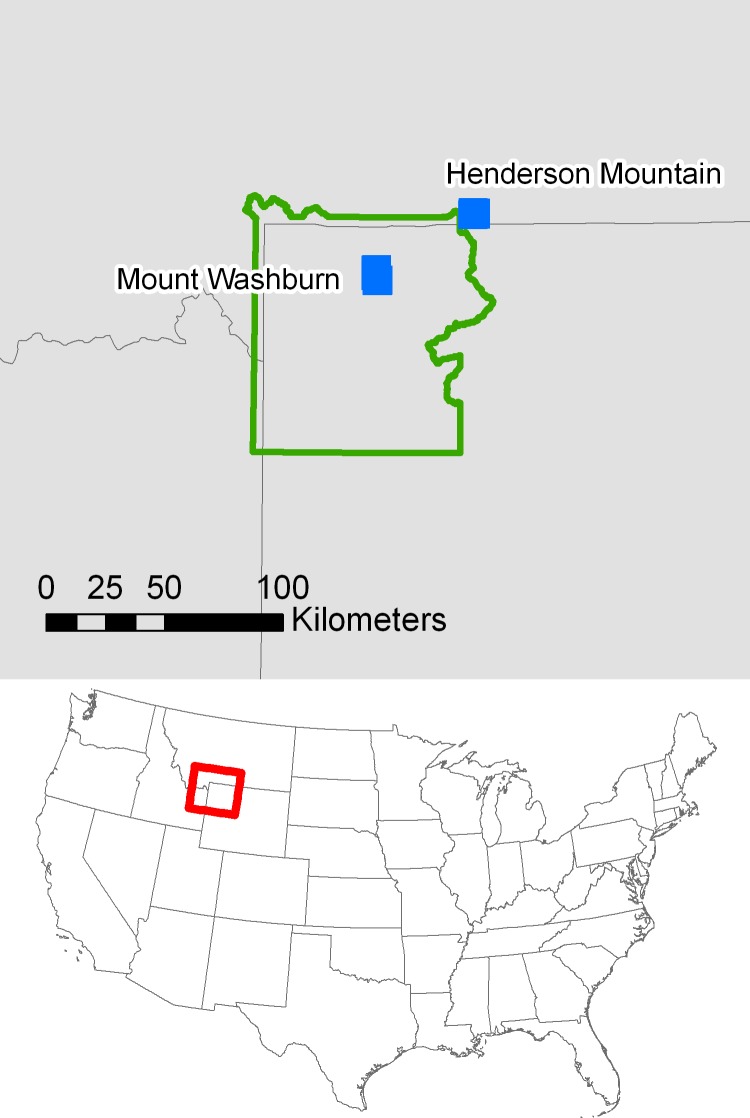
Map of sample collection locations. Top: Sample collection locations for *Pinus albicaulis* Engelm. (Pinaceae). Henderson Mountain, Custer Gallatin National Forest, MT and Mt. Washburn, Yellowstone National Park, WY. Bottom: Geographic location of Yellowstone National Park, Central Rocky Mountains.

In association with an on-going ecological study, and to compare with past population genetic research in this region, two populations were selected from the Greater Yellowstone Ecosystem for evaluation; Henderson Mountain (Custer Gallatin National Forest, MT, USA; letter approving sample collection) and Mt. Washburn (Yellowstone National Park, WY, USA; research permit YELL-2016-SCI-6064), which are separated by 50 km (Fig 1) [11]. Overstory composition in these two subalpine stands consists of cone-bearing *P*. *albicaulis*, *P*. *contorta* Douglas, *Picea engelmannii* Parry ex Engelm., and *Abies lasiocarpa* (Hooker) Nuttall. The two stands are 0.5–5.0 km from post-fire study areas established in the Greater Yellowstone Ecosystem in 1990, so the selected stands represent putative seed sources for areas regenerating following the 1988 Yellowstone fires. *Pinus albicaulis* from Mt. Washburn was collected within a 13.3 ha (0.13 km^2^) area ranging from 2,810–3,005 m elevation in north-central Yellowstone National Park, on north and west-facing slopes, 7–9 km north of Canyon Junction. Samples from Henderson Mountain were collected within a 15.7 ha (0.16 km^2^) area ranging from 2,690–2,785 m elevation on south and east-facing slopes, 3 km northeast of Cooke City, MT, and 5–7 km northeast of Yellowstone Park. For both populations, 20 individuals nearest to randomly generated points were sampled, with five fascicles collected from each individual and silica-dried for extended storage. Tree diameter was measured at breast height (DBH; S2 Table).

Eight individuals from Henderson Mountain were screened for initial locus testing, and 20 individuals from each population were used for final population genetic assessment. Due to minor amplification failure, a total of 26 individuals from Henderson Mountain and 22 individuals from Mt. Washburn were sampled to bring the total sample size up to 20 individuals for each of the 23 loci selected from the initial screening. Standard genetic diversity measures, Hardy-Weinberg Equilibrium, and inbreeding coefficients were calculated using *GenAlEx* 6.5 [71, 72]. The occurrence and frequency of null alleles was estimated using ML-NullFreq with 10,000 permutations [73]. Histograms of average number of alleles per locus were created in *R* [74] to visualize differences between populations. Population differences in the mean number of alleles per locus and expected heterozygosity are defined as the difference between Mt. Washburn and Henderson Mountain. Differences in means were plotted in *R*, along with 95% confidence intervals. Partitioning of population genetic variation was also evaluated by analysis of molecular variance (AMOVA) using Nei's genetic distances and 9,999 permutations (*GenAlEx* 6.5). Isolation-by-distance within and between populations was tested using Mantel’s correlation with 9,999 permutations in *GenAlEx* 6.5 [71, 72, 75].

## Results

Of the sequences screened, we identified 1,667 (1,341 di- and 326 tri-nucleotide) microsatellite-containing sequences from shotgun DNA libraries of *P*. *albicaulis*. Primer pairs were designed for 308 unique microsatellite-containing loci, and these were evaluated for PCR amplification success and segregation in a panel of diploid needle tissue. Of these markers, ten novel *P*. *albicaulis*-derived loci amplified consistently and cleanly and were polymorphic in the two screened populations (Tables 1 and 2). The total number of alleles (N_A_) for these novel loci ranged from 2–9 in the combined populations, with an average of 3.3 alleles per locus (Table 2). Observed heterozygosity (H_O_) ranged from 0.02–0.50 ($\bar{x}$ = 0.18) and expected heterozygosity (H_E_) ranged from 0.02–0.40 ($\bar{x}$ = 0.18). All loci conformed with Hardy–Weinberg Equilibrium (HWE) (Table 2).

**Table 1:** Descriptive information for the 10 microsatellite loci isolated from *Pinus albicaulis*.

Locus name	Primer sequence (5'–3')	Repeat motif	Allele size (bp)
ALBI_B08	F: CGTTACAGTAATTGGACG	(CG)_n_	259–271
	R: GTTTGTAGCAAACAGTAC		
ALBI_063	F: TCTCTTCGAGATTTAAACCCA	(AT)_n_	197–201
	R: ACCGTTCAGAATTAGACCAT		
ALBI_069	F: GATCTCCGACCGTAATGTGT	(AG)_n_	195–197
	R: AGTGCGACCTTACAAAGAGG		
ALBI_112	F: ATTTCCCACCATCCCTAATG	(TC)_n_	284–325
	R: GCGGACATACGGATCAAGT		
ALBI_113	F: ATGAGGACCAGGAGTAAAT	(AG)_n_	273–276
	R: GCTATTGTGTAAGCCTGCGT		
ALBI_116	F: AGAACATGAAAGGCCTAGGAA	(AT)_n_	157–159
	R: GGATCCTCTGGCCAAGTTAG		
ALBI_149	F: TAGGATCCCTTCTTTTTGGG	(AC)_n_	199–226
	R: TTATGTCTTGGCTTGGCAGT		
ALBI_151	F: GAACTTCTGAGGAAGCTTGATG	(AG)_n_	167–197
	R: TTCTGATCAATCTAAAGGTCAATCT		
ALBI_160	F: TGTTATTGCTAACGGAGATGG	(TG)_n_	229–233
	R: TTTTCTATTGTAGACAGTGTCGTCTT		
ALBI_171	F: ACAAAGCCACGAACAATCA	(CT)_n_	267–271
	R: GCCAATTGTTTGTTGGCTCTTTTAAC		

Locus name, primer sequences, repeat motif, and allele size range for the 10 microsatellite loci isolated for *Pinus albicaulis* Engelm. (Pinaceae). Annealing temperature is 58°C for all loci.

**Table 2:** Microsatellite loci, source species, and application to two populations of *Pinus albicaulis* in the Greater Yellowstone Ecosystem.

Locus name	Source species	N_a_			Henderson Mountain						Mt. Washburn					
			H_o_	H_e_	N_A_	A_P_	A_N_	H_O_	H_E_	HWE	N_A_	A_P_	A_N_	H_O_	H_E_	HWE
ALBI_B08	*P*. *albicaulis*	2	0.025	0.025	2	1	0.0	0.045	0.044	ns	1	0	0.3	0.000	0.000	-
ALBI_063	*P*. *albicaulis*	3	0.500	0.404	3	1	0.0	0.455	0.361	ns	2	0	0.0	0.550	0.439	ns
ALBI_069	*P*. *albicaulis*	3	0.075	0.073	3	2	0.0	0.150	0.141	ns	1	0	0.0	0.000	0.000	-
ALBI_112	*P*. *albicaulis*	3	0.132	0.169	2	0	0.0	0.105	0.100	ns	3	1	0.2	0.158	0.234	ns
ALBI_113	*P*. *albicaulis*	3	0.231	0.207	3	1	0.0	0.143	0.135	ns	2	0	0.0	0.333	0.278	ns
ALBI_116	*P*. *albicaulis*	2	0.024	0.024	2	1	0.0	0.048	0.046	ns	1	0	0.0	0.000	0.000	-
ALBI_149	*P*. *albicaulis*	9	0.370	0.360	6	2	0.0	0.346	0.308	ns	7	3	0.0	0.400	0.423	ns
ALBI_151	*P*. *albicaulis*	2	0.023	0.023	2	1	0.0	0.043	0.043	ns	1	0	0.0	0.000	0.000	-
ALBI_160	*P*. *albicaulis*	3	0.319	0.354	3	0	0.0	0.407	0.381	ns	3	0	0.1	0.200	0.304	ns
ALBI_171	*P*. *albicaulis*	3	0.150	0.224	3	0	0.3	0.150	0.296	ns	3	0	0.0	0.150	0.141	ns
**Mean**	** **	**3.3**	**0.184**	**0.184**	**2.9**	**0.9**	**0.00**	**0.189**	**0.185**	** **	**2.4**	**0.4**	**0.06**	**0.179**	**0.182**	** **
PcHJM	*P*. *cembra*	5	0.390	0.468	4	1	0.1	0.381	0.481	ns	4	1	0.0	0.400	0.446	ns
RPS119	*P*. *strobus*	7	0.375	0.774	6	1	0.4	0.350	0.794	***	6	1	0.2	0.400	0.710	ns
RPS124	*P*. *strobus*	4	0.184	0.218	3	0	0.3	0.111	0.202	ns	4	1	0.0	0.250	0.229	ns
RPS127	*P*. *strobus*	9	0.275	0.804	9	3	0.5	0.238	0.814	***	6	0	0.3	0.316	0.737	***
P5	*P*. *koraiensis*	7	0.289	0.771	6	2	0.4	0.300	0.743	***	5	1	0.2	0.350	0.745	***
P29	*P*. *koraiensis*	4	0.450	0.363	3	1	0.0	0.450	0.366	ns	3	1	0.0	0.450	0.359	ns
P37	*P*. *koraiensis*	7	0.475	0.697	5	1	0.3	0.350	0.690	***	6	2	0.1	0.600	0.699	**
P38	*P*. *koraiensis*	11	0.353	0.470	8	5	0.2	0.300	0.428	ns	5	2	0.1	0.200	0.271	*
P45	*P*. *koraiensis*	15	0.563	0.893	13	3	0.2	0.571	0.892	*	12	2	0.2	0.579	0.892	ns
P52	*P*. *koraiensis*	5	0.512	0.647	4	1	0.2	0.381	0.602	ns	4	1	0.0	0.650	0.681	ns
P62	*P*. *koraiensis*	3	0.487	0.489	3	1	0.0	0.474	0.517	ns	2	0	0.0	0.500	0.455	ns
P63	*P*. *koraiensis*	8	0.375	0.714	8	2	0.4	0.350	0.739	***	6	0	0.2	0.400	0.666	**
PisATG0012	*P*. *parviflora*	8	0.400	0.659	6	2	0.1	0.500	0.648	ns	6	2	0.2	0.300	0.648	**
**Mean**	** **	**7.2**	**0.394**	**0.602**	**6.0**	**1.8**	**0.25**	**0.369**	**0.614**	** **	**5.3**	**1.1**	**0.12**	**0.420**	**0.591**	** **

Source species for primer development, and genetic diversity for 10 microsatellite loci isolated for *Pinus albicaulis* Engelm. (Pinaceae) and 13 microsatellite loci transferred, based on 20 individuals from each population. (Henderson Mountain, Custer Gallatin National Forest, MT, and Mount Washburn, Yellowstone National Park, WY.)

N_a_ = total number of alleles; H_o_ = observed heterozygosity, total sample; H_e_ = expected heterozygosity, total sample; N_A_ = number of alleles; A_P_ = private alleles; A_N_ = null allele frequency; H_O_ = observed heterozygosity; H_E_ = expected heterozygosity; HWE = Hardy-Weinberg Equilibrium (ns = *p* > 0.05; * = *p* <0.05; ** = *p* < 0.01; *** = *p* < 0.001)

Although transferability of nSSR loci to the *P*. *albicaulis* genome transfer was highly variable depending on the source species, 13 of the 42 loci (27% overall) amplified successfully, yielding interpretable results (Tables 2 and 3). Sequencing revealed these nSSRs to be the expected basepair motifs, though they tended to have fewer repeats than listed for the source species. Loci developed for *P*. *koraiensis* had the highest rate of transferability, with eight of 13 (62%) tested loci amplifying in *P*. *albicaulis*, followed by *P*. *parviflora* with one of three (33%), and *P*. *strobus* with three of 19 (16%). Loci developed for *P*. *cembra* had the lowest rate of transferability with only one successful locus from a pool of 14 loci (7%). The average number of alleles per locus varied from 5–8 for the transferred microsatellites (Table 3). For transferred loci, N_A_ ranged from 3–15 ($\bar{x}$ = 7.2), H_O_ ranged from 0.18–0.56 ($\bar{x}$ = 0.39), and H_E_ ranged from 0.22–0.89 ($\bar{x}$ = 0.60). Eight loci deviated from HWE (Table 2).

**Table 3:** Results of microsatellite locus transferability to *Pinus albicaulis* for primers developed in other *Pinus* species.

Source species	Loci tested	Loci transferred	Success (%)	Average N_a_
*P*. *cembra*	14	1	7.1	5.0
*P*. *koraiensis*	13	8	61.5	7.5
*P*. *strobus*	19	3	15.8	6.7
*P*. *parviflora*	3	1	33.3	8.0

Number of loci tested per species, number that amplified in *Pinus albicaulis* Engelm. (Pinaceae), percent of total loci tested that transferred successfully, and average number of alleles (N_a_) per locus. Primers were screened in two populations (Henderson Mountain, Custer Gallatin National Forest, MT, and Mount Washburn, Yellowstone National Park, WY).

**Fig 2:**
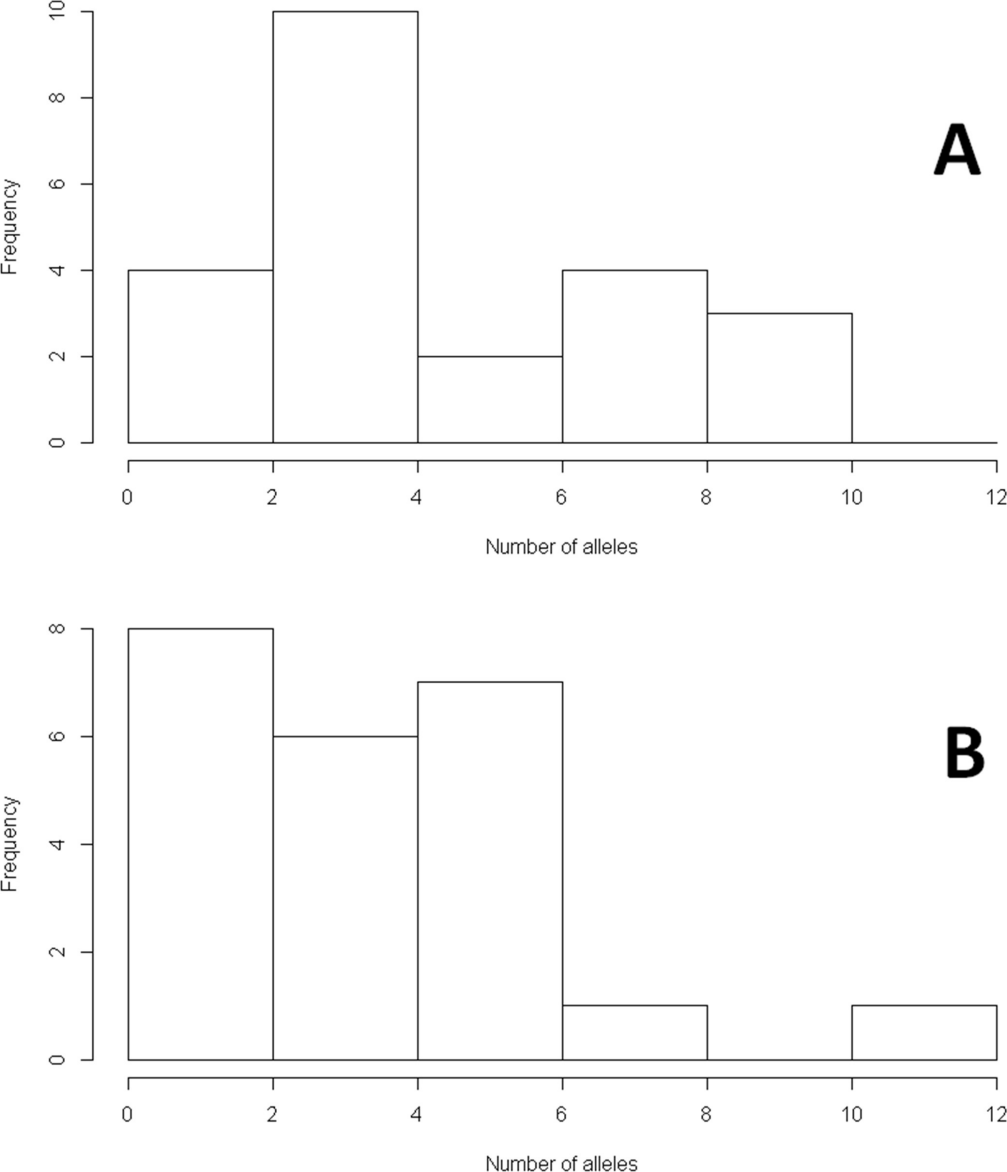
Number of alleles per locus in two *Pinus albicaulis* populations. Histograms of the count frequency per locus for number of alleles for all of 23 microsatellite loci for two populations of *Pinus albicaulis* Engelm. (Pinaceae) **A)** Henderson Mountain, Custer Gallatin National Forest, MT, and **B)** Mount Washburn, Yellowstone National Park, WY.

For our survey populations, the global range of alleles per locus was similar, but Henderson Mountain generally had more genetic diversity than Mt. Washburn (Fig 2, S1 Fig). Henderson Mountain had a higher average number of alleles, ranging from 2–9 ($\bar{x}$ = 4.6), with an average of 1.30 private alleles per locus. H_O_ ranged from 0.04–0.56 ($\bar{x}$ = 0.29), and H_E_ ranged from 0.04–0.84 ($\bar{x}$ = 0.43) (Table 2). N_A_ for Mt. Washburn ranged from 1–12 ($\bar{x}$ = 4.1), with an average of 0.91 private alleles per locus. H_O_ ranged from 0–0.65 ($\bar{x}$ = 0.32), and H_E_ ranged from 0–0.90 ($\bar{x}$ = 0.41) (Table 4). Eight loci deviated from Hardy–Weinberg equilibrium in both populations (Table 2). Null allele frequency estimates for transferred loci ranged from 0–0.51 at Henderson Mountain, and 0–0.30 at Mt. Washburn (Table 2).

**Table 4:** Genetic variability at microsatellite loci in two *Pinus albicaulis* populations.

	Average N_A_	Average A_P_	Average H_O_	Average H_E_	F_IS_
Novel	3.30		0.184	0.184	0.007
Transferred	7.15		0.394	0.602	0.291
Henderson	4.57	1.30	0.291	0.428	0.320
Washburn	4.09	0.91	0.315	0.413	0.236
Overall	4.33	1.11	0.303	0.427	0.168

Average number of alleles (N_A_), private alleles (A_P_), observed heterozygosity (H_O_), expected heterozygosity (H_E_) and inbreeding coefficient (F_IS_) by SSR development (novel developed for *Pinus albicaulis* Engelm. [Pinaceae], transferred developed in other pines) and population (Henderson Mountain, Custer Gallatin National Forest, MT, and Mount Washburn, Yellowstone National Park, WY).

Across all markers, the overall inbreeding coefficient (F_IS_) was 0.32 for Henderson Mountain, and 0.24 for Mt. Washburn. However, loci developed specifically for *P*. *albicaulis* showed a much lower inbreeding coefficient (0.007) than those transferred from other species (0.291) (Table 4); this difference is likely a consequence of the higher number of null alleles in the transferred SSR markers. An AMOVA identified 91% of variance within populations, and 9% among populations. The spatial distance between samples at Henderson Mountain was larger (range = 8–538 m, $\bar{x}$ = 231) than at Mt. Washburn (range = 7–348 m, $\bar{x}$ = 173) (S3 Table). Mantel’s test within populations showed no correlation between genetic and geographic distances at Henderson Mountain (r_x,y_ = -0.120, p = 0.121), indicating that individuals are no more closely related than expected with a random spatial pattern (S2 and S3 Figs). A weakly positive correlation at Mt. Washburn (r_x,y_ = 0.162, p = 0.051) indicated that individuals may be more closely related genetically to nearby individuals than expected under a random spatial pattern (S2 and S3 Figs). Between the two populations, there was no spatial autocorrelation (r_x,y_ = 0.029, p = 0.149) (S3 Table).

## Discussion

Our primary objective—the development of reliable nSSR markers useful for assessing genetic diversity in populations of *P*. *albicaulis*—was accomplished with the identification of ten novel and 13 transferred microsatellite loci. Of the 308 novel microsatellites identified from shotgun sequencing of *P*. *albicaulis*, only ten loci were sufficiently polymorphic and reliable to be identified for marker development, corresponding to a success rate of ~3%. This is approximately one-fifth the success rate of SSRs developed in *Chamaecyparis lawsoniana* (A. Murray) Parl. using these same methods [60]. This conversion rate is extremely low for species of genus *Pinus*, which have already been characterized by low conversion rates, attributed to the complexity of their genomes (> 20Gbp) and the presence of large numbers of paralogous SSR families [76], [77], [78]. The low yield is likely due to the selection of shorter motifs, which are known to show lower mutation rates than long microsatellite repeats [79]. The success rate of SSR marker transfer from other white pines to *P*. *albicaulis* was 13 out of 49—almost 27%. The average number of alleles per locus of the transferred microsatellites (7.2) is more than twice that of the novel microsatellites (3.2), suggesting that the transferred loci may be useful in studies requiring high individual identification power, or detecting differences in spatial differentiation. For studies sensitive to the presence of null alleles and error in heterozygote identification, the markers derived from *P*. *albicaulis* are likely to perform better, albeit at the cost of variability. Notably, transferability of loci from the two east Asian species (*P*. *koraiensis* and *P*. *parviflora*) was substantially higher than for the European (*P*. *cembra*) and North American (*P*. *strobus*) species tested. This study highlights the opportunity for screening newly published novel microsatellites from closely related species in *P*. *albicaulis*, such as recently developed for *P*. *sibirica* [80]. While these were not included in the present study, they also have a high likelihood of transferability, due to the close genetic affinity between *P*. *albicaulis* and *P*. *sibirica*.

Our secondary objective was to provide a case example of the utility of these microsatellites for population genetic research by comparing genetic diversity in two populations of *P*. *albicaulis* previously studied using allozyme analysis [43]. Despite the relatively small geographic distance between these sites (50 km), the nSSR markers revealed that both Henderson Mountain and Mt. Washburn had private alleles, averaging 1.3 and 0.9 per locus, respectively. This is likely a result of the small sample sizes (20 individuals per site) used in this study, though such sample sizes should yield adequate estimates [81]. Compared to other tools for measuring genetic diversity in *P*. *albicaulis*—including allozymes [43] and SNPs [47]—the microsatellite loci described here have a higher mean number of alleles per locus, as is expected with microsatellite markers. For example, with allozyme analysis these sites showed 1.6–1.7 alleles per polymorphic locus [43]. The surveyed SSRs revealed around three times the number of alleles per polymorphic locus, with 4.1–4.6 (Table 3).

All deviations from HWE are due to an excess of homozygous genotypes (not heterozygotes) and are most likely the result of allelic dropout (null alleles). All loci that violate HWE are transferred loci, which may indicate imperfect conservation of primer binding sites in *P*. *albicaulis*, or a presence/absence polymorphism in some chromosomal lineages. In these instances, null alleles fail to amplify, leading to decreased observed heterozygosity and apparent reaction failure in null homozygous individuals [82]. The potential impact of null alleles may be revealed from our F_IS_ values, which are large and positive for both populations, leading to potentially erroneous inferences of moderate inbreeding. Six loci—RPS119, RPS127, P5, P45, P63, and PisATG0012—were estimated to have high null allele frequencies in both populations. Loci that diverge from HWE and show evidence of null alleles should be used with caution as they inflate homozygosity and thus may not be applicable for all analyses (Table 2). Looking at only the novel loci developed for *P*. *albicaulis*, the inbreeding coefficient is very small, conforming to expectations from past research indicating random mating [43]. For studies sensitive to the presence of null alleles and error in heterozygote identification, the markers derived from *P*. *albicaulis* are less informative but may yield more accurate estimates.

Mantel's test revealed low genetic differentiation among populations and high gene flow, with no correlation between geographic distance and genetic relatedness among populations. Within populations, Mantel’s correlation shows no spatial autocorrelation at Henderson Mountain, with individuals no more or less closely related genetically to those nearby geographically than expected at random. There is weak evidence for isolation by distance in the Mt. Washburn population, with a positive Mantel correlation indicating that genetic distance between individuals increases with increasing physical distances. However, the AMOVA, which revealed that 9% of genetic differentiation occurred between populations, indicates greater population differentiation compared to SNP findings [47]. A larger sample of trees across varying distances will be required to determine the geographic distances required for isolation-by-distances and population differentiation.

Our results with *P*. *albicaulis* reveal that locus transferability is a viable option for developing microsatellites in five-needle pines, with the caveat that null alleles may show high frequencies in some interspecific comparisons. Other efforts in cross-species amplification in *Pinus*, however, have had mixed results, with some cases appearing successful [50, 83, 84], and others showing far lower success rates than novel marker development [67, 85]. It may be the case that the loci we tested for transfer were located at stable sites in the *P*. *albicaulis* genome (e.g., near centromeres, regions of low recombination, or in linkage with conserved genes).

These 23 nSSR markers provide a useful low-cost method to survey *P*. *albicaulis* genetic diversity, population structure, and parentage. Employing high-level PCR multiplexing [48] would further increase screening efficiency. Previous molecular studies have used isozymes and chloroplast DNA microsatellites to support the division of *P*. *albicaulis* into five “seed zones” in the Inland West [34]. Since molecular markers primarily reflect neutral processes (migration; drift), and the extent of differentiation of neutral markers is usually far lower than differentiation in adaptive traits [34], this approach by itself may be of limited value for developing seed zones that ensure seedlings are well-adapted to growing conditions at specific planting sites. Nevertheless, neutral markers like these SSRs offer a relatively rapid screen that can be used to partition genetic variation into genealogical groups, without the major expense of establishing and measuring multiple test plantations over multiple environments (e.g., [86]). These genealogical groups can be used to prioritize populations for blister rust resistance screening in *P*. *albicaulis*, and for the exploration of climate change mitigation strategies (e.g., [87, 88]). Since SSRs typically reveal neutral differentiation, these markers can also provide a baseline for identifying candidate SNPs that are associated with adaptive traits, such as cold tolerance [89], aridity traits, and wood property traits [90]. The identification of F_ST_ outliers depends upon accurate estimation of background differentiation and ancestry, and microsatellite markers are often the ideal choice for these estimates [91].

It is imperative to develop a predictive model for how much genetic diversity will remain in *P*. *albicaulis* populations as they decline in response to white pine blister rust infection and mountain pine beetle outbreaks, particularly with respect to those trees remaining as seed sources after disturbances. The time needed to recover genetic diversity following stand-replacing disturbances will be an essential research question as well, especially as time intervals between fires decrease as a result of climate change. The Greater Yellowstone Ecosystem in particular is projected to experience reduced intervals between wildfires, decreasing from the historical average of 100–500 years to fewer than 30 years by 2099 [5]. Given this trend, we will need to predict whether *P*. *albicaulis* will be able to recover genetic diversity rapidly enough to avoid bottlenecking.

Given the imperative conservation goals for *P*. *albicaulis*, SSR markers offer a complement to SNPs as a multi-allelic marker that can be used to measure the diversity—and changes in diversity—of rare genetic variation. Studies assessing genetic diversity based on these neutral SSR loci, and a more comprehensive sample of potentially-adaptive diversity (e.g., [45, 46]), can help guide future conservation efforts and restoration plans for *P*. *albicaulis*.

## Supporting information

S1 Fig Difference in number of alleles and heterozygosity between two *Pinus albicaulis* populations. **A)** difference in mean number of alleles (-0.74) and 95% confidence interval of the difference (-2.29 to 0.81) and **B)** difference in mean expected heterozygosity (-0.015) and 95% confidence interval of the difference (-0.178 to 0.148) of 23 microsatellite loci for two populations of *Pinus albicaulis* Engelm. (Pinaceae): Henderson Mountain, Custer Gallatin National Forest, MT, and Mount Washburn, Yellowstone National Park, WY. Plotted points are the difference in means, Washburn minus Henderson, and the lines are the 95% confidence intervals around those points.
(TIF)

S2 Fig Mantel's test frequency distributions in two *Pinus* albicaulis populations. Frequency distribution of random R_xy_ versus the observed R_xy_ (red line) for 9999 permutations, from Mantel’s test on two *Pinus albicaulis* Engelm. (Pinaceae) populations (**A**. Henderson Mountain, Custer Gallatin National Forest, MT, and **B**. Mount Washburn, Yellowstone National Park, WY). R_xy_ is the correlation between geographic and genetic distance between individuals. A positive value indicates individuals are more closely related genetically to those nearby geographically than random, and a negative value indicating individuals are less closely related to those nearby.
(TIF)

S3 Fig Correlation between geographic distance and genetic distance in two *Pinus albicaulis* populations. Correlation between geographic distance and genetic distance, from Mantel’s test on two *Pinus albicaulis* Engelm. (Pinaceae) populations. **A)** Henderson Mountain, Custer Gallatin National Forest, MT showing no correlation between genetic and geographic distances (rx,y = -0.120, p = 0.121) and **B)** Mount Washburn, Yellowstone National Park, WY showing a positive correlation (rx,y = 0.162, p = 0.051).
(TIF)

S1 Table Descriptive information for screened primers of microsatellites developed for other species of *Pinus*. Locus name, source species of primer development, and allele size range for 49 primers screened for transferability to *Pinus albicaulis* Engelm. (Pinaceae). Bold entries successfully transferred.
(DOCX)

S2 Table Diameter at breast height of two sampled *Pinus albicaulis* populations. Minimum, maximum, and mean diameter at breast height (DBH) in centimeters compared between *Pinus albicaulis* Engelm. (Pinaceae) sampled at two populations (Henderson Mountain, Custer Gallatin National Forest, MT, and Mount Washburn, Yellowstone National Park, WY).
(DOCX)

S3 Table Distances between sampled *Pinus albicaulis* within two populations in the Greater Yellowstone Ecosystem. Minimum, maximum, and mean distance between *Pinus albicaulis* Engelm. (Pinaceae) sampled at two populations (Henderson Mountain, Custer Gallatin National Forest, MT, and Mount Washburn, Yellowstone National Park, WY). R_xy_ and P_(rxy-rand > = rxy-data)_ from Mantel’s correlation, showing isolation by distance and spatial autocorrelation, testing for each population and for all individuals combined.
(DOCX)
