# Supplementary figures and images for: Development of nuclear microsatellite loci for Pinus albicaulis Engelm. (Pinaceae), a conifer of conservation concern
Source: PLoS One. 2018 Oct 18;13(10):e0205423. doi: 10.1371/journal.pone.0205423 (PMC6193661; doi:10.1371/journal.pone.0205423)

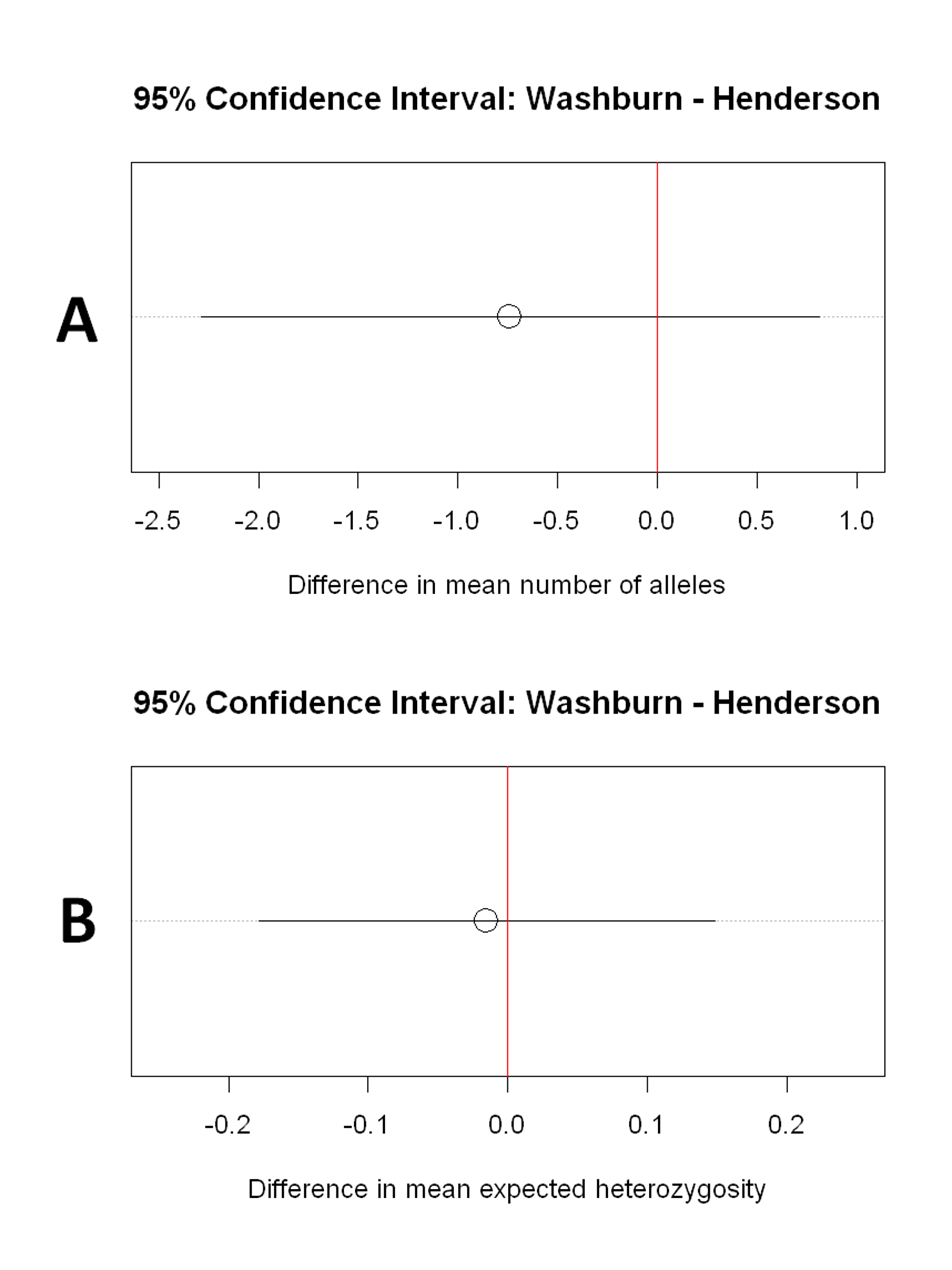

Supplement: S1 Fig — A) difference in mean number of alleles (-0.74) and 95% confidence interval of the difference (-2.29 to 0.81) and B) difference in mean expected heterozygosity (-0.015) and 95% confidence interval of the difference (-0.178 to 0.148) of 23 microsatellite loci for two populations of Pinus albicaulis Engelm. (Pinaceae): Henderson Mountain, Custer Gallatin National Forest, MT, and Mount Washburn, Yellowstone National Park, WY. Plotted points are the difference in means, Washburn minus Henderson, and the lines are the 95% confidence intervals around those points. (TIF) [file pone.0205423.s001.TIF]

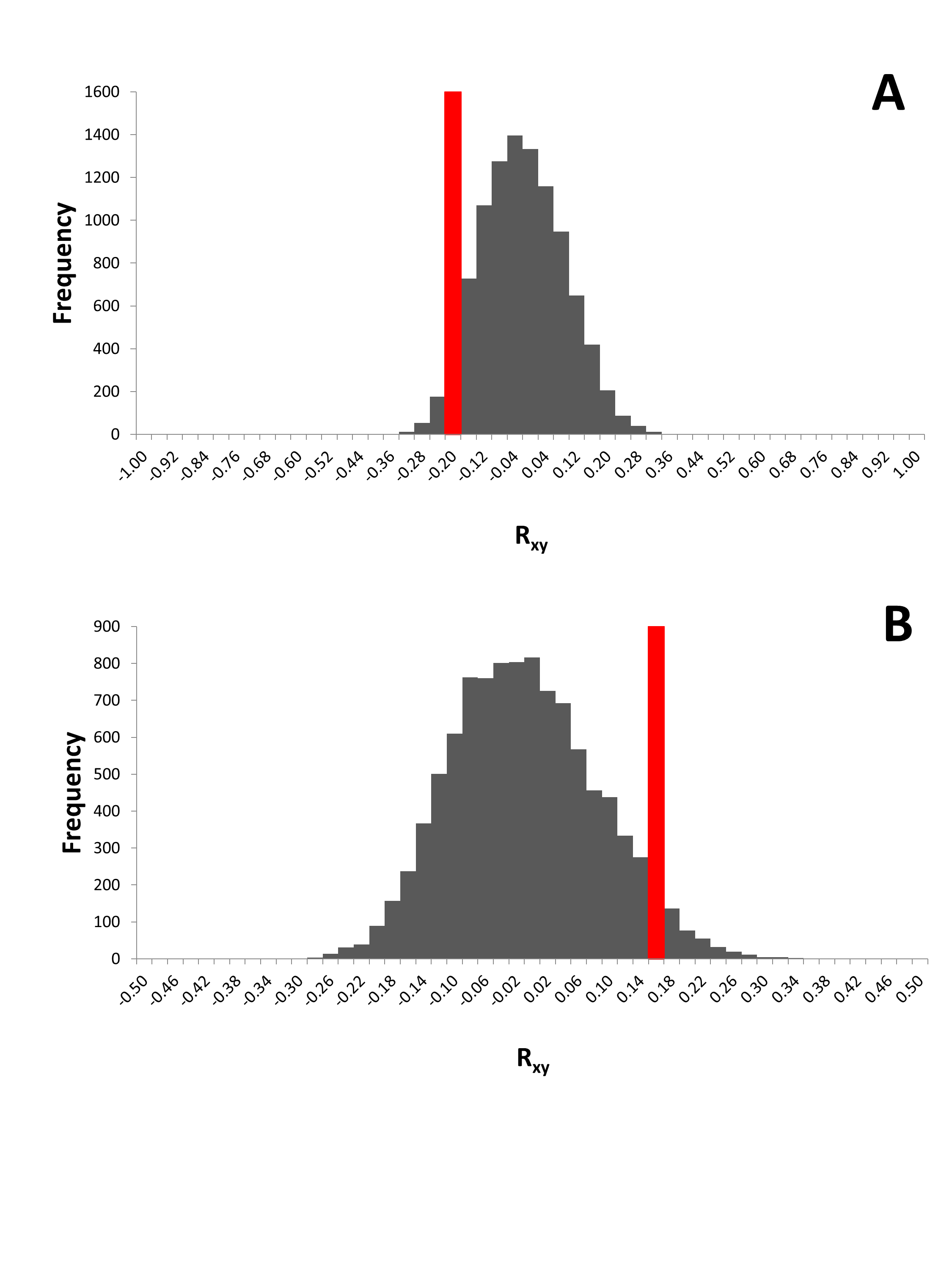

Supplement: S2 Fig — Frequency distribution of random Rxy versus the observed Rxy (red line) for 9999 permutations, from Mantel’s test on two Pinus albicaulis Engelm. (Pinaceae) populations (A. Henderson Mountain, Custer Gallatin National Forest, MT, and B. Mount Washburn, Yellowstone National Park, WY). Rxy is the correlation between geographic and genetic distance between individuals. A positive value indicates individuals are more closely related genetically to those nearby geographically than random, and a negative value indicating individuals are less closely related to those nearby. (TIF) [file pone.0205423.s002.TIF]

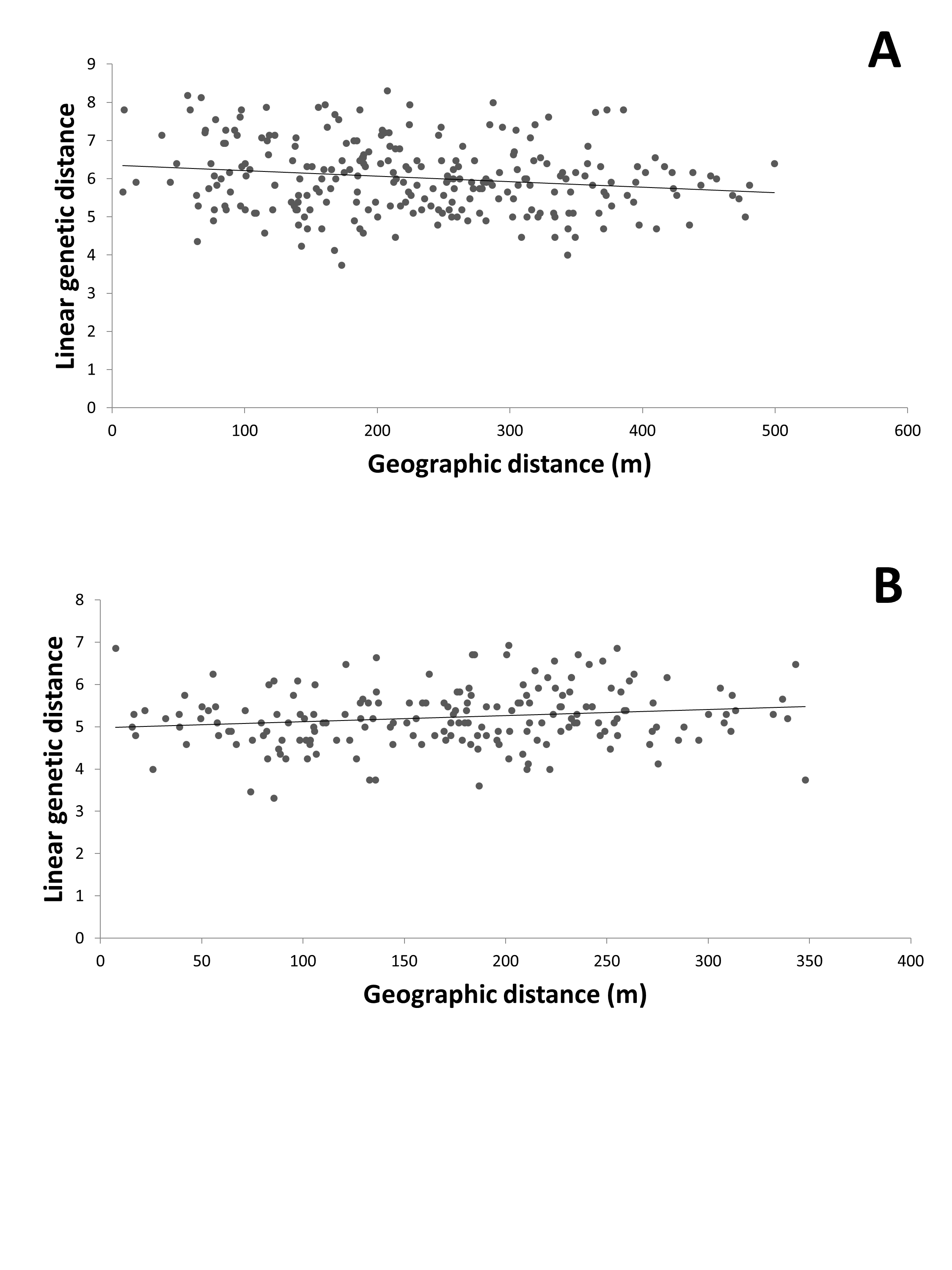

Supplement: S3 Fig — Correlation between geographic distance and genetic distance, from Mantel’s test on two Pinus albicaulis Engelm. (Pinaceae) populations. A) Henderson Mountain, Custer Gallatin National Forest, MT showing no correlation between genetic and geographic distances (rx,y = -0.120, p = 0.121) and B) Mount Washburn, Yellowstone National Park, WY showing a positive correlation (rx,y = 0.162, p = 0.051). (TIF) [file pone.0205423.s003.TIF]
