# Supplementary material for: Development of nuclear microsatellite loci for Pinus albicaulis Engelm. (Pinaceae), a conifer of conservation concern
Source: PLoS One. 2018 Oct 18;13(10):e0205423. doi: 10.1371/journal.pone.0205423 (PMC6193661; doi:10.1371/journal.pone.0205423)
Supplement: S1 Table — Locus name, source species of primer development, and allele size range for 49 primers screened for transferability to Pinus albicaulis Engelm. (Pinaceae). Bold entries successfully transferred. (DOCX) [file pone.0205423.s004.docx]

**S1 Table**

| Locus  name | Source species | Expected size (bp) |  | Locus  name | Source species | Expected size (bp) |
| --- | --- | --- | --- | --- | --- | --- |
| Pc1b | *P. cembra* | 166–202 |  | RPS1b | *P. strobus* | 207 |
| Pc3 | *P. cembra* | 195–269 |  | RPS2 | *P. strobus* | 163 |
| Pc7 | *P. cembra* | 342–384 |  | RPS6 | *P. strobus* | 162 |
| Pc18 | *P. cembra* | 152–156 |  | RPS12 | *P. strobus* | 185 |
| Pc22 | *P. cembra* | 345–375 |  | RPS18 | *P. strobus* | 160 |
| Pc23 | *P. cembra* | 221–257 |  | RPS20 | *P. strobus* | 138 |
| Pc25 | *P. cembra* | 123–138 |  | RPS25b | *P. strobus* | 107 |
| Pc35 | *P. cembra* | 162–165 |  | RPS34b | *P. strobus* | 145 |
| **PcHJM** | ***P. cembra*** | **168–188** |  | RPS39 | *P. strobus* | 172 |
| PcBUG | *P. cembra* | 101–133 |  | RPS50 | *P. strobus* | 170 |
| PcYAU | *P. cembra* | 144–162 |  | RPS60 | *P. strobus* | 269 |
| Pc28Z | *P. cembra* | 288–324 |  | RPS84 | *P. strobus* | 147 |
| PcCQG | *P. cembra* | 169–179 |  | RPS90 | *P. strobus* | 164 |
| PcSWK | *P. cembra* | 236–318 |  | RPS118b | *P. strobus* | 160 |
| **P5** | ***P. koraiensis*** | **120** |  | **RPS119** | ***P. strobus*** | **205** |
| P6 | *P. koraiensis* | 125 |  | **RPS124** | ***P. strobus*** | **149** |
| P16 | *P. koraiensis* | 160 |  | **RPS127** | ***P. strobus*** | **193–237** |
| **P29** | ***P. koraiensis*** | **145** |  | RPS150 | *P. strobus* | 248 |
| **P37** | ***P. koraiensis*** | **154** |  | RPS160 | *P. strobus* | 246 |
| **P38** | ***P. koraiensis*** | **160** |  | Pis0198 | *P. parviflora* | 285–321 |
| **P45** | ***P. koraiensis*** | **173** |  | **PisATG0012** | ***P. parviflora*** | **167–210** |
| P51 | *P. koraiensis* | 204 |  | Pis0235 | *P. parviflora* | 143–198 |
| **P52** | ***P. koraiensis*** | **138** |  |  |  |  |
| P53 | *P. koraiensis* | 205 |  |  |  |  |
| **P62** | ***P. koraiensis*** | **134** |  |  |  |  |
| **P63** | ***P. koraiensis*** | **242** |  |  |  |  |
| P66 | *P. koraiensis* | 159 |  |  |  |  |
