# Supplementary material for: Development of nuclear microsatellite loci for Pinus albicaulis Engelm. (Pinaceae), a conifer of conservation concern
Source: PLoS One. 2018 Oct 18;13(10):e0205423. doi: 10.1371/journal.pone.0205423 (PMC6193661; doi:10.1371/journal.pone.0205423)
Supplement: S2 Table — Minimum, maximum, and mean diameter at breast height (DBH) in centimeters compared between Pinus albicaulis Engelm. (Pinaceae) sampled at two populations (Henderson Mountain, Custer Gallatin National Forest, MT, and Mount Washburn, Yellowstone National Park, WY). (DOCX) [file pone.0205423.s005.docx]

**S2 Table**

| Population | Minimum DBH | Maximum DBH | Mean DBH |
| --- | --- | --- | --- |
|  |  |  |  |
| Washburn | 3.2 | 57.0 | 17.2 |
| Henderson | 3.6 | 59.8 | 21.7 |
