# Supplementary material for: Development of nuclear microsatellite loci for Pinus albicaulis Engelm. (Pinaceae), a conifer of conservation concern
Source: PLoS One. 2018 Oct 18;13(10):e0205423. doi: 10.1371/journal.pone.0205423 (PMC6193661; doi:10.1371/journal.pone.0205423)
Supplement: S3 Table — Minimum, maximum, and mean distance between Pinus albicaulis Engelm. (Pinaceae) sampled at two populations (Henderson Mountain, Custer Gallatin National Forest, MT, and Mount Washburn, Yellowstone National Park, WY). Rxy and P(rxy-rand > = rxy-data) from Mantel’s correlation, showing isolation by distance and spatial autocorrelation, testing for each population and for all individuals combined. (DOCX) [file pone.0205423.s006.docx]

**S3 Table**

| Population | Minimum Distance | Maximum Distance | Mean Distance | R_xy_ | P |
| --- | --- | --- | --- | --- | --- |
| Washburn | 7.4 | 348.0 | 173.0 | 0.162 | 0.051 |
| Henderson | 8.0 | 537.8 | 230.6 | -0.120 | 0.121 |
| Overall | |  |  | 0.029 | 0.149 |
